# Design and development of a novel instrument for characterizing the mechanical properties of ex vivo human skin

**DOI:** 10.1038/s41598-026-42371-9

**Published:** 2026-03-10

**Authors:** Bastien Blanchard, Francis Ehrenfeld, Anthony Laffore, Corinne Nardin, Christophe Derail

**Affiliations:** Universite de Pau et des Pays de l’Adour, CNRS, IPREM, Pau, 64000 France

**Keywords:** Characterization and analytical techniques, Skin models

## Abstract

The viscoelastic properties of human skin are fundamental to its ability to maintain structural integrity. These mechanical characteristics are crucial for understanding skin behavior and have several important applications: (i) documenting the natural evolution of skin over time; (ii) providing an objective method to evaluate the efficacy of medical or dermo-cosmetic treatments; and (iii) identifying and quantifying pathological changes that affect the mechanical properties of the tissue. To address these needs, a novel instrument has been developed specifically to assess the viscoelastic properties of ex vivo human skin explants, maintained in physiological-like conditions over seven days. This device, described herein, enables both conventional tensile testing and dynamic mechanical analysis. The latter allows for the characterization of the skin’s two primary mechanical components—elasticity and energy dissipation—across a range of frequencies, thereby enabling the differentiation of these phenomena. Initial studies focused on repeatability and reproducibility using skin explants, confirming the robustness of both the instrument and the measurement protocol for ex vivo skin characterization. A comprehensive example of mechanical analysis is presented to illustrate the capabilities of the device. Ultimately, this instrument offers a promising approach for evaluating the viscoelastic behavior of human skin explants and for monitoring the impact of various stressors on skin mechanics over time.

**Supplementary Information:**

The online version contains supplementary material available at 10.1038/s41598-026-42371-9.

## Introduction

Skin is the organ at the interface between the outside and inside of the body, forming a protective envelope. It has to be highly resistant, flexible whatever the directions and to be able to return to its original state under all circumstances. The properties and characteristics of this organ, which is a functional biointerface, are therefore essential to ensure for instance protection of the body against mechanical, biological, and chemical aggressions^1^.

The skin is anatomically made up of three tissue layers which are, from the outer surface to the inner side of the body: the epidermis, the dermis, and the hypodermis. As each layer is a system that is itself made up of several parts, the structure of human skin is notoriously known for its complexity^2^.

According to various authors^3–9^, the dermis is the thickest and most important layer of the skin providing structure and mechanical strength. The dermis and its fibre networks largely control the deformation behaviour of the skin. It is a dense collagenous tissue in the general sense described as a heterogeneous structure composed of a few cells and of an extracellular matrix. This matrix is composed of collagen fibres of various types, elastic fibres and of the ground substance. The dermis is therefore a composite structure in the mechanical sense: a combination of collagen fibres and elastin fibrils surrounded by a host hydrogel-like material (the ground substance), thus playing the main role in ensuring the mechanical integrity function of the skin. This complex structure allows for a global complex mechanical behaviour which is necessary to ensure effective resistance to the many mechanical aggressions of daily life.

The mechanical properties of the skin are of great importance in many clinical and cosmetic applications. They have therefore been extensively studied experimentally both ex vivo and in vivo^10–12^. Although individual fibers exhibit predominantly a linear mechanical behaviour, their collective arrangement within the tissue leads to a transition toward a non-linear behaviour when subjected to mechanical strain of approximately 30%^13,14^. In addition to the type of structure, the density of the collagen fibres generally play a key role on the global mechanical properties of the tissue^15^.

Being the skin a multi-functional and multi-component organ, a complex range of mechanical properties are generated. Indeed, the skin is characterised by a non-homogeneous, non-linear, viscoelastic, and anisotropic behaviour with a natural pre-stress^3,16^.

The anisotropy of the human skin was predicted in 1861 by Langer^17^ and demonstrated in 1966 by Daly^18^. this property of skin, and more generally that of all soft collagenous tissues, can be observed by performing an uni-axial quasi-static tensile test to observe the associated typical stress-strain curve. Under tensile load, the overall response of the skin in extension is characterised by a J-shape stress-strain curve that could be described as bilinear in the elastic domain (or even hyperelastic), in which each phase is interpreted by the role played by each of the families of fibres^19^. Indeed, elastic fibres provide the elasticity to the skin at the beginning of traction and are responsible for its low elastic modulus during the first phase of traction (phase of tensioning of elastic fibres and orientation of collagen fibres to resist traction), whereas collagen fibres resist large deformations after unfolding and reorientation in the direction of traction (phase of stretching of collagen fibres). The stiffer collagen fibres contribute to the high elastic modulus of the skin in the second tensile phase.

Skin, like all soft collagenous tissues, is a viscoelastic material: it has both elastic and viscous characteristics^16^. The viscoelasticity of the skin can be attributed to several factors, such as:

- + Fluid flows and the intrinsic viscoelasticity of the ground substance^13,16^;
- + The intrinsic viscoelasticity of collagen fibres. Minns^20^ showed the rate-dependent behaviour of collagen;
- + Dissipative friction between fibres and mesh rearrangement^21^. Guimberteau^22^ observed in vivo a phenomenon of sliding and reorganisation of the fibres, contributing to the dissipation of energy during stress.

Skin reveals different mechanical characteristics depending on its orientation: it is anisotropic. The first discovery of the anisotropy of human skin was made by Dupuytren^23^, who observed that circular wounds evolve into ellipses over time. This discovery was further investigated by the Austrian anatomist Karl Langer^17^ in 1861. He made circular incisions in cadavers to study the natural tension and anisotropy of skin. He found that by drilling these circular holes in the skin of a corpse, the resulting wounds were ellipsoidal and always oriented in the same direction at a given location in the body, revealing the existence of a pre-stressed state. By making several incisions close together, he was able to delineate and map lines of tension, called “Langer lines”.

These biomechanical analyses of the skin are fundamental to quantify and qualify the behaviour of a tissue. It is an essential element to facilitate the development of tissue engineering and the development of certain medical devices^24^. Measuring the mechanical properties of the skin is generally of threefold interest^25^:

- + to obtain a description of the natural history of the skin over the years, in particular during growth and ageing phases;
- + to have a method of objective evaluation of the effect of certain medical or cosmetic products;
- + to observe and quantify skin pathologies that can lead to an alteration of the mechanical properties, such as scleroderma, collagenosis or hyperkeratosis for example.

The most commonly used techniques described in the literature are traction, suction, bulge, and indentation^26,27^; and some of these techniques have led to commercialised devices. The skin tensile test is widely used and is a standard test with which the skin is stretched parallel to its surface with a constant stretching speed^8,21,28–31^. Several research devices have been developed but only one device performing these extension tests has been commercialised: the Extensometer^®^ (Cutech). The suction test consists in applying, perpendicular to the skin and for a few seconds, a vacuum within a suction chamber glued to the skin surface^36–40^. Several suction devices are commonly used by researchers and clinicians, including the Dermaflex^®^ (Cortex Technology) and the Cutometer^®^ (Courage & Khazaka) which use an optical system for skin surface elevation measurements^41^. Among the various methods of mechanical testing, indentation testing is also used for determining the mechanical behaviour of skin in vivo. It consists of applying a load (pressure force) to the skin through an indenter and measuring the indentation it produces on the tissue surface^42–46^.

The different mechanical tests all provide relevant information characterising the behaviour of the skin and their interest lies in the need to multiply the type of loading to take into account the structural complexity of the tissue. In fact, each of them gives the skin’s response to a type of stress and characterises a particular feature of its behaviour under mechanical stress.

However, experiments are usually carried out in vivo, with the inherent errors of direct measurement on a poorly defined part of the body for a mechanical experiment and of biological variability due to statistics on a limited number of individuals. These in vivo tests are realistic because they are carried out directly on the living human body, but they are complex to set up experimentally and do not allow invasive and destructive measurements to be made. Ex vivo measurements, on the other hand, allow many mechanical parameters to be explored while isolating the skin in order to characterise only this tissue. In addition, ex vivo conditions enable to obtain samples that can be modified or attacked under severe chemical, biological and mechanical stress. These conditions therefore allow to follow the evolution of the skin’s mechanical properties over time and under the possible effect of different stresses It is obvious that each of these approaches will present positive and negative outcomes for appropriate interpretation.

In this context, a specific instrument for evaluating the viscoelastic properties of human skin ex vivo has been developed^47^. This new device enables to characterise classical traction behaviour at constant traction strain rate, as well as the spectromechanical analysis to evaluate viscoelastic components, elasticity and dissipation at different frequencies, in order to discriminate each phenomenon. These different dynamic measurements provide a specific and original vision by measuring the overall viscoelastic properties and in particular by taking into account the dissipation parameters, which have been very little explored otherwise^48,49^. The proposed approach is to carry out spectromechanical analyses in order to establish a phenomenological link between the mechanical properties and the structure of the skin, with an approach analogous to the mechanical characterization of polymer materials. Moreover, this device allows to analyse explants which are maintained under physiological-like conditions over seven days.

The aim of these initial experiments carried out in this study is and reported herein, on the one hand, to prove that the instrument and the test methods employed are robust for measuring the mechanical properties of an ex vivo human skin explant maintained under physiological like conditions over seven days, and on the other hand, to describe a complete mechanical characterisation of an ex vivo human skin explant using this new equipment, as described below.

Overall, the specific advantages of the described instrument are the testing conditions, i.e., ex vivo yet in physiological-like conditions. Unlike most devices described in the literature, non-destructive dynamic tests (in the elastic range) can be conducted as pads adhere to the skin explant, otherwise maintained “alive” in its nutritive medium. Measurements can hence be repeated several times, over long time periods (up to seven days) and be subjected to various external stresses. Besides, different mechanical parameters of ex vivo human skin can be monitored over time as the combination of classical tensile and dynamic measurements enables characterization of both mechanical components of skin, elasticity and dissipation, across different frequencies. This spectrometry type measurement, over a wide frequency range, allows access to previously unexplored mechanisms of the skin’s viscoelastic behavior, which is in contrast to most other devices, which operate at fixed frequencies. It may also be worth insisting on the fact that we can control the geometry of the explants, and therefore achieve tighter control over the final values of the measured parameters, unlike devices used directly on patients (such as the cutometer^®^). Eventually, compared with indentation-based approaches, we can highlight complementarity: indentation provides very localized measurement, whereas our approach yields a more global mechanical assessment—offering complementary perspectives. This device, when used with skin explants, allows direct relevance to cosmetic and/or medical applications. Indeed, we can evaluate over time the impact of different stresses or treatments on the mechanical properties of the skin. Monitoring the various mechanical parameters and their evolution in response to treatments and stresses, under physiological conditions over several days, opens the door to significant scientific impact in fields such as dermatology (e.g., tracking the evolution of mechanical properties during a medical treatment).

## Results and discussion

### Repeatability and reproducibility tests

Before presenting the results that can be achieved using this new instrumentation, two kinds of preliminary studies were carried out in order to ascertain that the characterisation of ex vivo skin explants under physiological-like conditions is correct and accurate.

These tests allow the experimental method developed and described in the “Methods” section to be validated.

- Repeatability

**Fig. 1:**
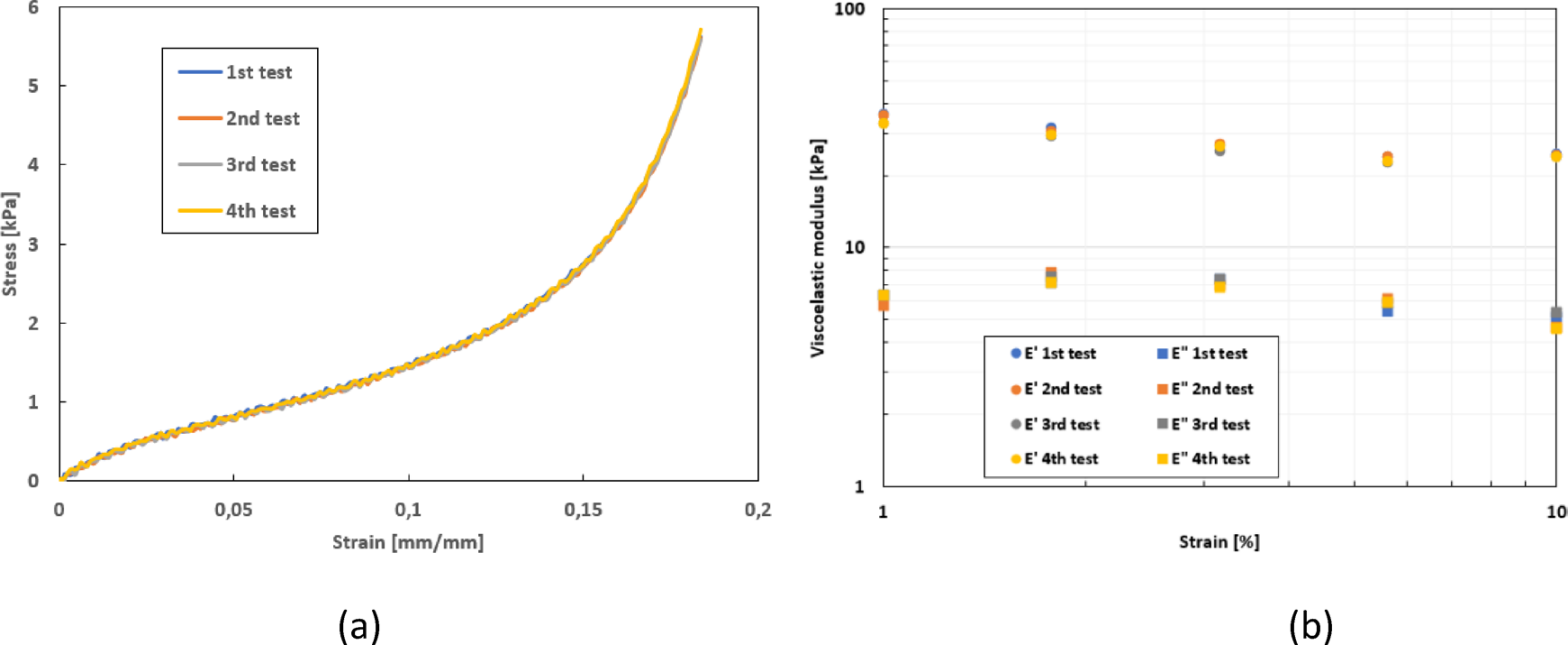
Repeatability of tests on a human skin explant. (**a**) Tensile test (speed = 10 mm min^-1^: 4 tests + average curve with standard deviation. (**b**) Dynamic test (strain sweep at 0.5 Hz): 4 tests + average curve with standard deviation.

Repeatability tests were carried out through both dynamic and quasi-static mechanical tests (Fig. 1).

To check the repeatability, the mechanical tests were carried out four times consecutively. As observed in Fig. 1, the curves for the quasi-static and dynamic tests are superimposed, demonstrating that the tests carried out with this device are repeatable in these two loading modes. This repeatability of the mechanical tests is attributable to the quality of the instrumentation and to the initial pre-conditioning of the skin, which stabilised the mechanical properties of the explant beforehand. These tests also showed that the skin explants did not deteriorate at strain levels below 20%.

- Reproducibility

**Fig. 2:**
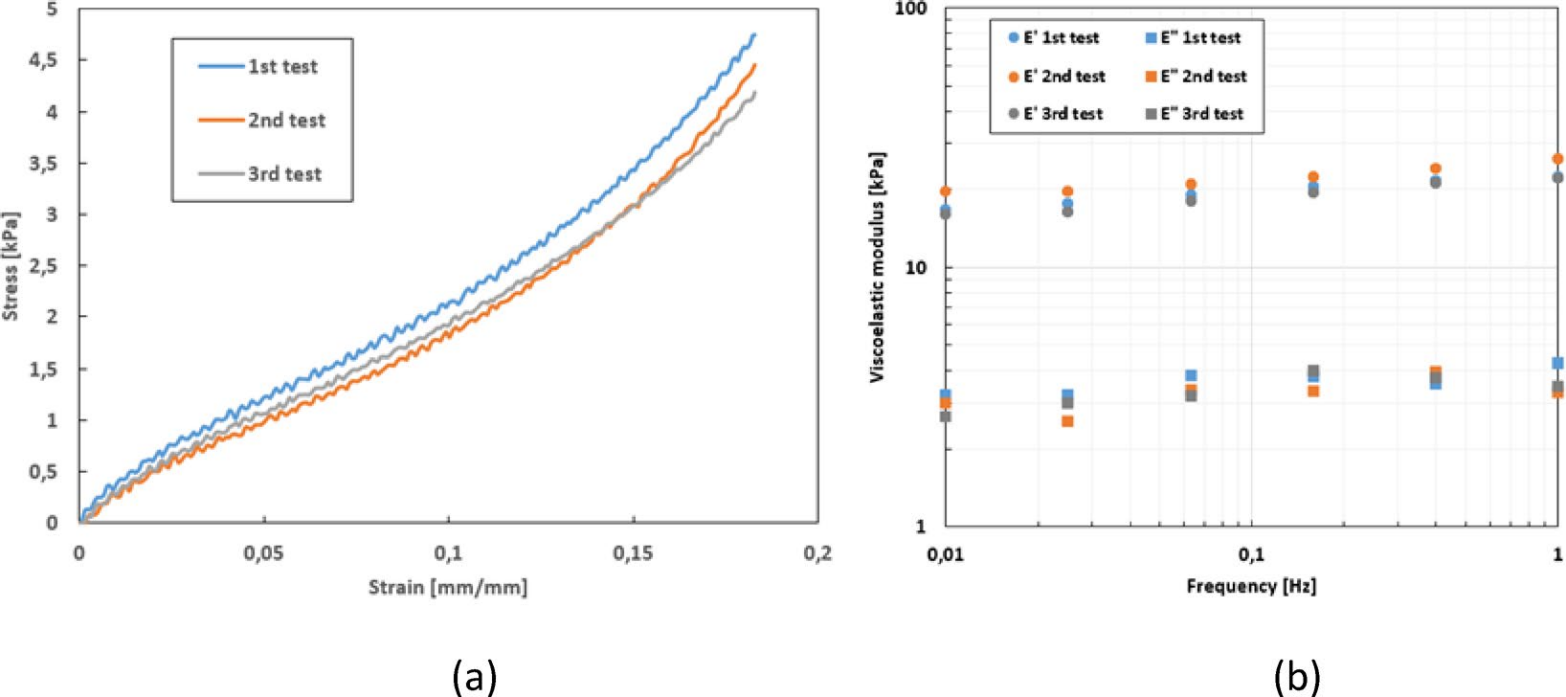
Reproducibility tests on a human skin explant. (**a**) Reproducibility in the tensile test mode (speed = 10 mm min^– 1^: 3 tests + average curve with standard deviation. (**b**) Example of reproducibility of a dynamic test (frequency sweep at 10%): 3 tests + average curve with standard deviation.

Reproducibility tests were carried out with dynamic and quasi-static mechanical tests (Fig. 2).

To check the reproducibility of the tests, the mechanical tests were therefore carried out several times consecutively, placing back the skin explant for each test. In other words, after the first test, the explant was completely removed from the device and then replaced for the second test, and so on. The curves for the quasi-static and dynamic tests show only slight variations. Less than 5% variation in simple tensile stress measurements and less than 10% variation in dynamic modulus measurements demonstrate that the tests carried out with this equipment are reproducible. This reproducibility of the mechanical tests is attributable to the quality of the instrumentation and the method developed for placing the skin explant in the equipment, i.e. placing the skin on a sample holder that automatically rises to bring it into contact with glued studs attached to the measuring instrument. These tests indicate that the procedure for placing the skin in the device is reproducible.

### Complete mechanical characterisation of an ex vivo human skin explant

Once all the preliminary results have been taken into account, the mechanical properties of an ex vivo human skin sample could be characterised in a comprehensive and reproducible way using this new measuring device. A complete and detailed characterisation of the mechanical properties of an ex vivo skin explant is presented and described in this Section.

**Fig. 3:**
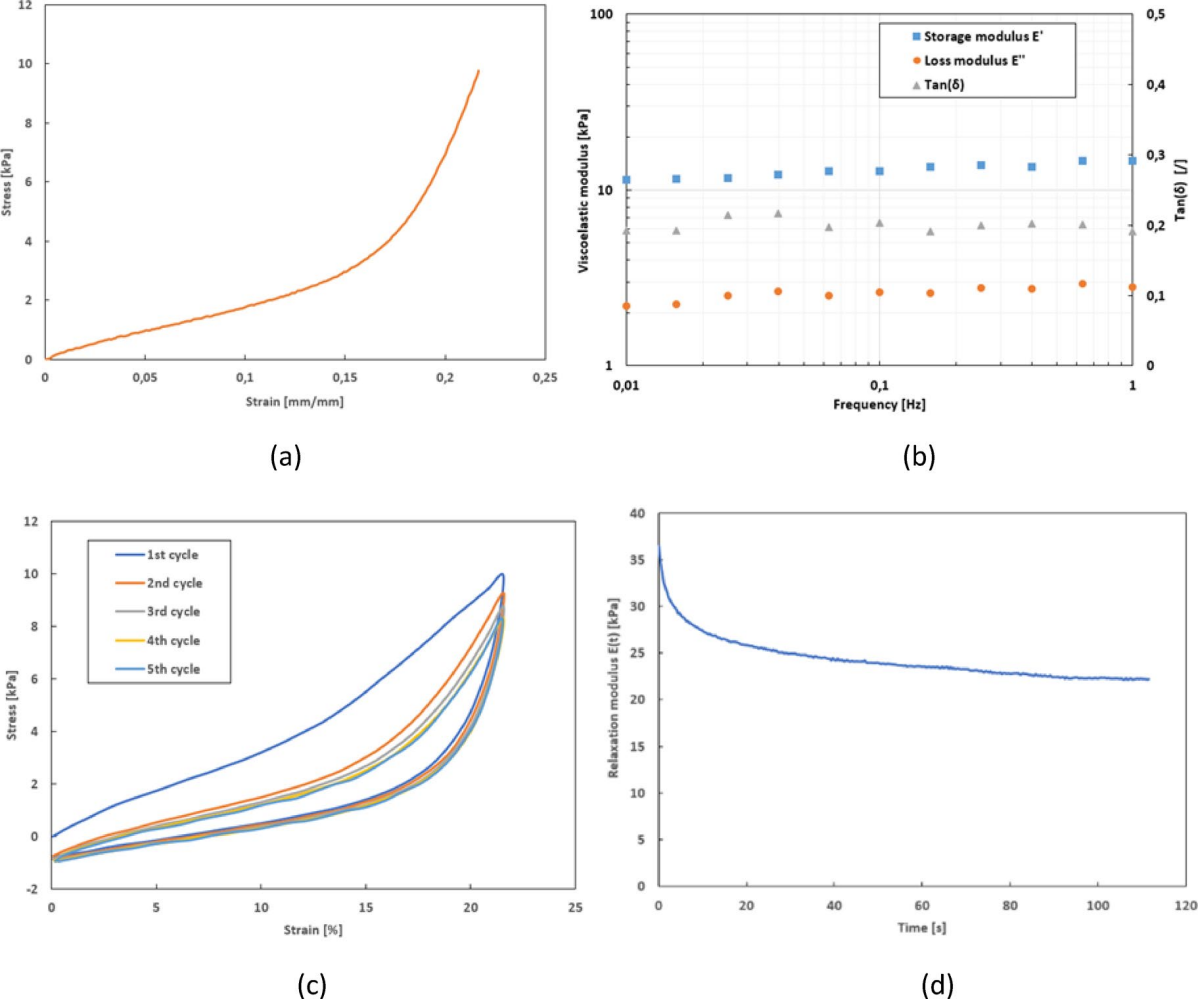
Representative results of all the different mechanical tests that can be carried out with the device to characterize an ex vivo skin explant. (**a**) Tensile test - stress versus strain graph (speed = 10 mm min^– 1^. (**b**) Frequency sweep test - graph of viscoelastic moduli, and loss factor, as a function of frequency (strain = 10% - T = 21 °C). (**c**) Cyclic loading-unloading test - stress versus strain graph (strain = 22% / speed = 10 mm min^– 1^. (**d**) Stress relaxation test - graph of relaxation modulus $\frac{\sigma\left(t\right)}{{\varepsilon}_{0}}$ as a function of time (with $\sigma\left(t\right)$ the stress at time$t$ and ${\varepsilon}_{0}$ the constant strain / tension = approx. 2 min / strain = 18%).

Conventional tensile tests show the global mechanical behavior of the skin explant under traction (Fig. 3.a). The ex vivo skin explants reveal a linear elastic mechanical behavior at the lower strains followed by a hardening phase. This behavior can even be considered as bilinear over the range of imposed strain. One can extract two moduli of elasticity of the skin: the Young’s modulus E_0_ at initial deformation and the modulus of elasticity at maximum deformation. The apparent Young’s modulus E_0_, measured at the origin of the stress-strain curves, is (28.0 ± 0.5) kPa. The modulus at maximum deformation is (88.4 ± 0.5) kPa, demonstrating the rapid stiffening of the explant, which is characteristic of the bilinear elastic behavior of the skin. The moduli of elasticity found in the literature^3,6,18,21,50–52^ are quite broad, ranging from a few kilopascals to hundreds of megapascals. The moduli measured by this new device are comparable and fall within a fairly low range of moduli of elasticity. Because of the way we attach the skin (glue) and our limited deformation range (less than 20%), which avoids damaging the explants during testing, we do characterize the low range of mechanical properties of human skin explants.

Spectromechanical analysis show that the storage modulus E’ is greater than the loss modulus E’’, whatever the frequency is, demonstrating the prominence of the skin’s elasticity properties (Fig. 3b). For the skin explant, the storage modulus and the loss modulus increase with the frequency. The elastic modulus E’ of the explant increases from (11.4 ± 0.5) kPa at 0.01 Hz to (14.7 ± 0.5) kPa at 1 Hz. The loss modulus E’’ of the explant increases from (2.2 ± 0.5) kPa at 0.01 Hz to (3 ± 2) kPa at 1 Hz. The slope of the storage modules E’ as a function of frequency is 0.75, whereas that of the dissipation modules E’’ as a function of frequency is 0.14. This means that as the test frequency increases, the mechanical strength of the skin also increases. Likewise, the higher the frequency, the faster the material must respond to strain.

The loss factor of the skin, i.e. tan(δ), is relatively constant at the different frequencies with an approximate value of 0.2 for this human skin explant, demonstrating the preponderance of the elastic part in the mechanical behavior of the skin.

The load-unload cycles enable, firstly, to evaluate and observe the stability of the mechanical response of the skin and, secondly, to evaluate the phenomenon of hysteresis with a skin explant. Figure 3c shows the evolution of the dissipated energy as a function of the load-unload cycle, and gives example of superimposed curves for five cycles. These results show the importance of the notion of pre-conditioning for soft tissues: the mechanical properties are stabilized at around the 5th cycle. The dissipated energy, calculated at the 5th loading-unloading cycle so that the mechanical behavior of the explants is stabilized, is (6.6 ± 0.7) µJ.

When the deformation of the material is kept constant, due to its viscoelastic nature, the skin dissipates energy to gradually return to a more stable state. Figure 3d shows that the stress in the skin, linked to the relaxation modulus, decreases over time until it reaches a plateau: this is the stress relaxation phenomenon. From the first few seconds of being held under tension, until about 30 s, the skin relaxes rapidly. The relaxation modulus drops from (36.5 ± 0.5) kPa at t_0_ to (25.0 ± 0.5) kPa after 30 s of constant mechanical tension. The skin then relaxes slowly and progressively over time. This measurement enables to measure the viscoelastic properties of the explant, and in particular to calculate the relaxation rates, which is (39 ± 3) % within the specific range of the test parameters used.

All the results that can be obtained using this new instrumentation are summarized in Table 1, showing the broad spectrum of mechanical analysis that is achievable.

**Table 1:** Biomechanical parameters of the ex vivo skin explant.

Mechanical test	Traction		Frequency scanning		Hysteresis cycles	Stress relaxation
Mechanical parameter	Young’s modulus E_0_ (kPa)	Modulus at 18% deformation (kPa)	Viscoelastic modulus at 0.01 Hz (kPa)	Viscoelastic modulus at 1 Hz (kPa)	Dissipated energy (µJ)	Relaxation rate (%)
Explant	28.0 ± 0.5	88.4 ± 0.5	E’: 11.4 ± 0.5	E’: 14.7 ± 0.5	6.6 ± 0.7	39 ± 3
			E’’: 2.2 ± 0.5	E’’: 3 ± 2		

## Discussion

Firstly, the tensile test provides information on the elastic behaviour of the skin. This elasticity arises from the extension of the various fibre networks which constitute the skin, resulting in a J-shape stress stretch curve. This form of curve is characteristic of the mechanical behavior of the skin^53^. From a physiological point of view, this typical shape of the curve with human skin is due to the various forces generated by the deformation of the network of the elastic and collagen fibers composing the dermis of the skin^19^. In the first region of skin stretch, at low rates of deformation, a strong extension is observed under low loading. This initial linear rise in the curve is mainly due to the resistance to stretching of the elastic network of elastic fibres in the dermis. During this phase, elastic fibres, which are highly extensible and easy to stretch, elongate. The collagen fibres, on the other hand, are coiled and wavy. The initially wavy collagen fibre bundles reorient and align along the loading axis. This is the transition phase when the skin’s mechanical behavior becomes stiffer: stress and rigidity increase. The last region is a new linear region corresponding to the stabilization of stiffness. This region reflects the elastic modulus of the collagen fibres, which are aligned in the direction of traction and offer maximum resistance.

Whereas elastic fibers are primarily responsible for the skin’s suppleness and resistance to small deformations, collagen is responsible for maintaining the skin mechanical integrity at larger deformations^6^. The skin therefore is characterized by a non-linear elastic behaviour (J-shape stress stretch curve), also known as hyperelasticity, which is often modelled in the literature by hyperelastic behaviour models^27,54,55^.

From spectromechanical analysis we observe that as the frequency increases, the two viscoelastic moduli increase slowly.

Actually, the high-frequency data represents the short-term behavior of the sample, where the local displacements of the natural polymer chains are sensed. Within these short loading times, little movement is possible within the material, the storage modulus is high and the skin behaves more rigidly. Conversely, at low frequencies, the data reflect the long-term behavior of the sample properties, over which the global displacements of the polymer chains occur. The lower the frequency, the greater the response time of the skin; the fibre networks are stressed on a larger length scale. When the loading time is longer, the sample has more time to react to the deformation and local movements of the chain become possible. Viscoelastic moduli are lower due to the adaptation of the tissue, and in particular of the fibre networks in the dermis, to relatively slow deformations.

As the frequency increases, the elasticity part, and the loss one both increase in equal proportions, and the loss factor is relatively constant. It should also be noticed that this factor is less than 1, confirming that the skin bhaves like a solid viscoelastic material (E’ > E’’). The viscoelastic behaviour of the skin is therefore clearly demonstrated with this type of mechanical test.

Under cyclic loading and unloading, viscoelastic materials such as skin exhibit hysteresis, which leads to dissipation of mechanical energy. From the 2nd cycle onwards, the mechanical behaviour of the explant is more or less constant, before stabilising completely around the 5th cycle. In fact, the stabilization of the mechanical behaviour of the explants occurs during the first cycles, when the energy dissipated is reduced and stabilized. The hysteresis phenomenon in skin explants represents the difference between the effort measured during loading and the effort measured during unloading. The amount of mechanical energy dissipated corresponds to the area between the loading phase and the unloading phase, within the hysteresis loop. The hysteresis cycle shows that the skin needs more force during an extension stress than during a contraction stress. When the skin is mechanically repositioned to its original position, it requires less force to relax. Energy is therefore dissipated in the form of heat between these two phases. The hysteresis phenomenon will be more or less marked but will always be present for skin explants, like for rubber^56^. This phenomenon is due to the viscoelastic behaviour of the skin, and can be linked to the friction forces between the constitutive fibres.

Finally, like the cyclic loading-unloading, the stress-relaxation test is used to assess the skin’s energy-dissipative behaviour. In contrast to dynamic loading, the mechanical test here consists in imposing a transient and static load on the explants. Relaxation is a non-instantaneous property: it needs a certain amount of time for the stress to reach its final value. Due to its viscoelastic nature, the skin dissipates energy and gradually returns to a more stable state when maintained under tension. To provide a physical explanation for the phenomena observed, we can assume that macroscopic relaxation reflects microscopic changes in the configurations of the macromolecular chains, i.e. the fibres constitutive of the material. Viscoelastic phenomena originate from the possibility of intra- or intermolecular movements of the chains to return to a position of statistical equilibrium. We assume that when the fibers are stressed when the skin is held under tension, they will reorient themselves along the axis of tension but will also be able to relax and allow the explants to return to a more stable state of tension. The fibers slide according to each other in the matrix. The more the skin relaxes by releasing stress, the more energy will be dissipated.

## Conclusion

Tests carried out on human skin explants, maintained under physiological-like conditions over seven days, have validated the potential of the instrument developed for measuring mechanical properties. To verify the reliability and accuracy of this new equipment, the first tests carried out were repeatability and reproducibility tests on the skin explants. These experiments using the device show that the configuration of the instrumentation and the measurement methods used are optimal and enable the mechanical properties of human skin to be accurately characterized in physiological-like conditions. Indeed, the tests carried out with this new device are repeatable and reproducible. The robustness of the device is therefore demonstrated. It gives close results even in the presence of slight variations in experimental conditions that may occur with the procedure used. Therefore, the equipment can be used by any user while guaranteeing reproducible and consistent results.

The peculiar approach proposed with this device is to perform spectromechanical analysis, in addition to other conventional mechanical tests, on a skin explant during the same measurement. All the mechanical tests that can be carried out provide a complete characterization of the mechanical properties of ex vivo human skin explants under tension. By controlling the displacement arms over a precise range of frequencies, it is possible to measure the mechanical properties of the skin over a wide range of loading times in order to characterize the overall behavior of the skin. In contrast to the speed-controlled loadings normally used to analyze the mechanical properties of a biological tissue, we are proposing a measurement system based on loadings over a range of frequencies, enabling to determine the complex modulus of the skin as a function of different frequencies in a single measurement phase. This type of measurement provides information on the viscoelasticity of the skin: the real part of the complex module characterizes the elastic properties whereas its imaginary part characterizes the dissipation properties.

In addition to the classic tensile mechanical tests, widely described in the literature, we bring new insights by measuring the overall rheological properties of the skin, and in particular by taking into account the dissipation parameters. To the best of our knowledge this enables to carry out an energy analysis that has never been reported on ex vivo skin explants kept in physiological-like conditions over several days.

All these results show that this device and the test method used are robust for characterizing the mechanical properties of the skin and that it could contribute to answering open questions in the dermatological field and could help in the analysis of the effects of different stresses on the mechanical properties of the skin. Indeed, this patented technology^47^ combined with the use of ex vivo skin samples under physiological-like conditions for several days, will allow the study of the effect of different chemical, biological and physical stresses on the mechanical properties of the skin.

## Methods

### Design of the experimental device

**Fig. 4:**
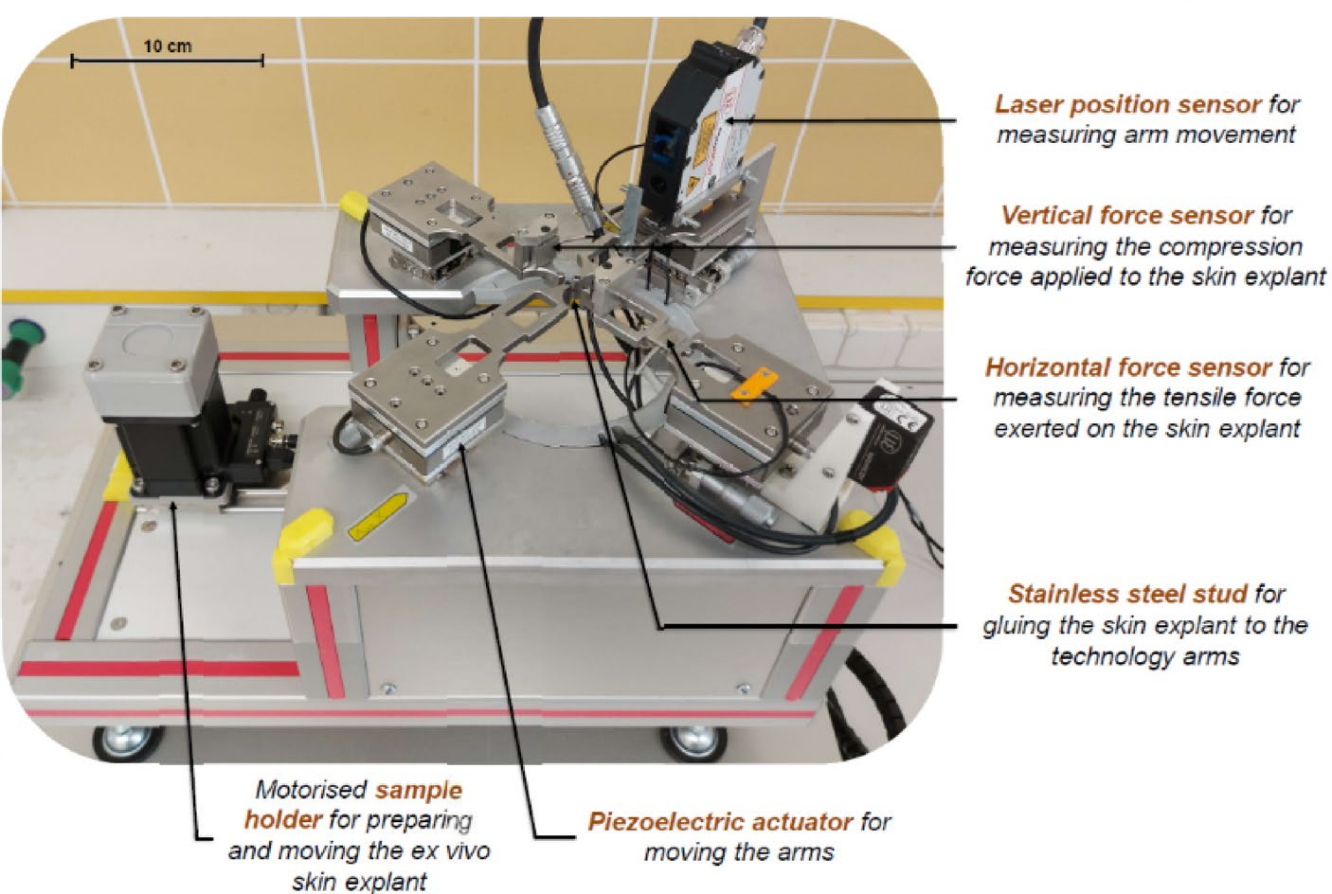
Picture and description of the current version of the device.

Figure 4 shows a picture of the device. As the patent indicates, it is a system for measuring the mechanical properties of a skin sample^47^. This device is designed to characterize the mechanical properties of an ex vivo skin sample held in a fixed position on a nutritional medium that maintains the skin sample in physiological-like conditions for at least seven days^57^. The principle is based on the application of a specific, controlled mechanical stress to the skin sample. This mechanical stress is applied in the form of traction in one direction in the plane of the skin and, eventually, at different frequencies or at different traction speeds. The mechanical responses of the stressed skin are then measured by various sensors.

Four mechanical loading modules are arranged around the centre of the device and each configured to exert a tensile force in a radial direction parallel to the surface of the skin sample. The translation arms are aligned in pairs and the axial displacement devices are synchronised to simultaneously displace two traction elements along the common axis. Each mechanical loading module comprises a translation arm connected:

- on the one hand, to the traction elements: the studs.
- and on the other hand, to a translational displacement system capable of displacing this arm: a piezoelectric nano-positioning table fixed to a manual micrometric displacement table.

The X-axis is equipped with a position sensor that accurately measures the position of a translation arm during its movement, and a tensile force sensor that measures the force exerted by the studs. This axis is used to assess the mechanical properties of the skin.

The Y-axis is equipped with these two sensors and an additional vertical force sensor to measure the compression force exerted by the studs. In this work, this axis is used to initially place the skin sample on the instrument using the vertical force sensors. The motorised sample holder is positioned on a mobile plate that allows the skin sample to be moved around the measurement zone, under the four mechanical loading modules. A camera is also configured to observe and record the deformation zone of the skin surface.

The entire system comprises the measuring device and a control and calculation unit to manage the whole equipment. This unit includes a control program (LabVIEW^®^ program), which controls the displacement components (piezoelectric displacement tables) of the measuring device in order to move the translation arms. The traction force sensors, position measurement sensors and imaging equipment are also connected to this unit. All the signals measured by the sensors are then processed in this unit to calculate the mechanical properties of the skin. Various types of tests, including quasi-static tests and dynamic tests, enable the mechanical properties of the skin to be comprehensively evaluated.

### Test protocol and ex vivo skin explant

The software interface allows the operator to follow a well-defined procedure to place an ex vivo skin explant in the instrumentation and to perform a mechanical test on this skin explant. The main steps in this procedure are described here.

The skin is first placed on the motorised sample holder, which is then positioned in the centre of the device. The end of the two studs of the X measurement axis is then glued using a liquid cyanoacrylate adhesive. The sample holder rises automatically to bring the skin into contact with the glued studs, and stops when the compression force entered in the software and measured on the Y-axis studs is reached. Once the adhesive has dried and solidified (in a few minutes), the mechanical tests can be carried out.

Positioning the studs is an important step, as it determines how well the mechanical tests are carried out. Precise positioning of the skin, guaranteed by vertical force sensors and a motorised sample support, ensures reproducible measurements.

Once the mechanical tests have been completed, the lift support is lowered to its initial position with the studs still glued to the surface of the skin. The studs are then removed and peeled off by inducing a slight flexion in the skin at the adhesive point. At the end, the explant is placed back in the incubator.

The human skin explants used in this work come from the Eurofins BIO-EC laboratory. Explants, also called organ-cultured skin or skin organ, are small skin samples taken from humans after surgical interventions (mainly in the abdomen), according to legal restrictions. These are therefore round skin biopsies of human origin, prepared from surgical skin residues, also known as skin plasty, donated by adult donors with their consent and collected in compliance with all applicable regulations. The diameter of the explants can vary from 12 to more than 40 mm; this diameter is defined by the operator, according to the studies to be carried out, who uses a punch to cut the skin plasty. The thickness of the explants varies from 2 to 3 mm, depending on their morphology. After decontamination, re-sizing, and fat cell removal (de-hypodermisation: only the dermis and epidermis are preserved), the skin explant is deposited on a specific appropriate culture medium. Thus, it can be studied over the course of a few days; we therefore speak of “ex vivo” skin, which is kept alive like “in vivo” skin.

For the tests, these skin explants are removed from an incubator, where they are placed in specific environmental and culture conditions (37 °C with 5% of CO_2_ and 95–98% of humidity), and positioned in the instrumentation. All mechanical tests are performed within a limited timeframe to ensure optimal survival of the explant and to quickly return it to the incubator. All tests are completed in approximately 30 min; they are carried out at a constant temperature, which is the ambient temperature in the test room: around 21 °C.

### Mechanical testing

- Due to the structural composition of biological soft tissues, their intrinsic viscoelastic behaviour produces uneven mechanical results when the tissue is subjected to mechanical loading for the first time^58^. Preconditioning is therefore considered as a necessary step to overcome the effects of soft tissue manipulation and establish a reproducible set of experiments. Such a procedure allows the tissues to reorganise themselves in order to adapt gradually to the load and therefore to obtain more consistent data during mechanical testing. Each characterised skin explant was therefore subjected to loading cycles in order to precondition the sample before carrying out the full tests. In general, the skin explants used required between three and ten cycles of uniaxial tensile stress before reaching the preconditioned state. Here, ten loading cycles were required to stabilise the mechanical behaviour of the skin and avoid what we call the Mullins effect.
- The repeatability testing method consists of placing a skin explant, following the procedure described above, and performing a mechanical test on the same skin explant several times in a row. This means that the same skin sample is analysed by the same operator, using the same measurement procedure and method, all in the shortest possible time.
- The reproducibility testing method consists of placing a skin explant, following the procedure described above, and performing a mechanical test before removing it; the whole process is repeated several times consecutively.

The notion of reproducibility is very important for ex vivo studies, as experiments can be carried out at different times on a skin explant. For example, to monitor the mechanical properties of the explant over time or to subject it to stress, the explant must be removed and replaced in an incubator for its preservation between experiments. This process of removing and reinstalling the sample can lead to errors in the mechanical tests.

- The tests that can be carried out to characterise human skin using the instrumentation are as follows:
  - Tensile test (skin deformation at a constant tensile speed),
  - Dynamic mechanical analysis (DMA) (skin sinusoidal deformations of constant amplitude and decrease in test frequency),
  - Loading-unloading cycles,
  - Stress relaxation test (constant instantaneous skin deformation over a period of time).
- It is important to note that in our case, we do not observe any significant extensions that would lead to plastic and irreversible deformation of the skin, at which the fibre networks would be broken.

## Supplementary Information

Below is the link to the electronic supplementary material.

Supplementary Material 1

Supplementary Material 2

## Data Availability

The datasets used and/or analyzed will be made available from the corresponding author on reasonable request: Christophe Derail should be contacted to request the data.

## References

[1] Krutmann, J., Bouloc, A., Sore, G., Bernard, B. A. & Passeron, T. The skin aging exposome. J. Dermatol. Sci. 85, 152–161 (2017). 10.1016/j.jdermsci.2016.09.015 27720464

[2] McGrath, J. A. & Uitto, J. Anatomy and Organization of Human Skin. in Rook’s Textbook of Dermatology (eds. Burns, T., Breathnach, S., Cox, N. & Griffiths, C.) 1–53 (Wiley-Blackwell, Oxford, UK, 2010).

[3] Agache, P. Physiologie de la peau et explorations fonctionnelles cutanées. Ed. *Médicale Int.* (2000).

[4] Diridollou, S. et al. An in vivo method for measuring the mechanical properties of the skin using ultrasound. *Ultrasound Med. Biol.* **24**, 215–224 (1998). 9550180 10.1016/s0301-5629(97)00237-8

[5] Dunn, M. G. & Silver, F. H. Viscoelastic Behavior of Human Connective Tissues: Relative Contribution of Viscous and Elastic Components. Connect. *Tissue Res.* 12, 59–70 (1983). 10.3109/03008208309005612 6671383

[6] Silver, F. H., Freeman, J. W. & DeVore, D. Viscoelastic properties of human skin and processed dermis. *Skin Res. Technol.* 7, 18–23 (2001). 10.1034/j.1600-0846.2001.007001018.x 11301636

[7] Ventre, M., Mollica, F. & Netti, P. A. The effect of composition and microstructure on the viscoelastic properties of dermis. *J. Biomech.* **42**, 430–435 (2009). 19159885 10.1016/j.jbiomech.2008.12.004

[8] Delalleau, A. Analyse du comportement mécanique de la peau in vivo. (Université de Saint-Etienne, 2007).

[9] Crichton, M. L. et al. The viscoelastic, hyperelastic and scale dependent behaviour of freshly excised individual skin layers. *Biomaterials* 32, 4670–4681 (2011). 10.1016/j.biomaterials.2011.03.012 21458062

[10] Xu, F., Lu, T. J. & Seffen, K. A. Biothermomechanical behavior of skin tissue. *Acta Mech. Sin.* 24, 1–23 (2008).

[11] Pissarenko, A. & Meyersa, M. A. The materials science of skin_ Analysis, characterization, and modeling. *Prog Mater. Sci.* **110**, 100634 (2020).

[12] Arnold, N., Scott, J. & Bush, T. R. A review of the characterizations of soft tissues used in human body modeling: Scope, limitations, and the path forward. *J. Tissue Viability* 32, 286–304 (2023). 10.1016/j.jtv.2023.02.003 36878737

[13] Hendriks, F. Mechanical Behavior of Human Skin in Vivo - a Literature Review. Nat.Lab. Unclassified report 820. *Philips Res. Laboratories* (2001).

[14] Brèque, C. Développement et mise en oeuvre de méthodes optiques pour la mesure de relief et de champ de déformations en vue de la modélisation d’organes biologiques. (Université de Poitiers, 2002).

[15] Roeder, B., Kokini, K., Sturgis, J., Robinson, J. & Voytik-Harbin, S. Tensile Mechanical Properties of Three-Dimensional Type I Collagen Extracellular Matrices With Varied Microstructure. *J. Biomech. Eng.* **124**, 214–222 (2002). 12002131 10.1115/1.1449904

[16] Wilkes, G., Brown, I. & Wildnauer, R. The biomechanical properties of skin. *CRC Crit. Rev. Bioeng.* **1**, 453–495 (1973). 4581809

[17] Langer, K. On the anatomy and physiology of the skin. I. The cleavability of the cutis. (Translated from Langer. sitzungsbericht der mathematisch-naturwissenschaftlichen classe der kaiserlichen academie der wissenschaften, 44, 19, 1861). *Br. J. Plast. Surg*. 31, 3–8 (1978). 342028

[18] Daly, C. H. Biomechanical Properties of Dermis. *J. Invest. Dermatol.* 79, 17–20 (1982). 10.1111/1523-1747.ep12544620 7086188

[19] Brown, I. A. A scanning electron microscope study of the effects of uniaxial tension on human skin. *Br. J. Dermatol.* **89**, 383–393 (1973). 4759952 10.1111/j.1365-2133.1973.tb02993.x

[20] Minns, R. J., Soden, P. & Jackson, D. The role of the fibrous components and ground substance in the mechanical properties of biological tissues: a preliminary investigation. *J. Biomech.* **6**, 153–165 (1973). 4632628 10.1016/0021-9290(73)90084-5

[21] Delalleau, A., Josse, G., Lagarde, J. M., Zahouani, H. & Bergheau, J. M. Characterization of the mechanical properties of skin by inverse analysis combined with an extensometry test. *Wear* **264**, 405–410 (2008). 10.1016/j.jbiomech.2005.05.001 15990103

[22] Guimberteau, J. C. et al. Introduction à la connaissance du glissement des structures sous-cutanées humaines. *Ann. Chir. Plast. Esthét*. **50**, 19–34 (2005). 15695007 10.1016/j.anplas.2004.10.012

[23] Dupuytren, G. Traité théorique et pratique des blessures par armes de guerre. (1834).

[24] Payne, A. P. Measurement of properties and function of skin. *Clin. Phys. Physiol. Meas.* **12**, 105–129 (1991). 1855358 10.1088/0143-0815/12/2/001

[25] Tran, H. V. Caractérisation des propriétés mécaniques de la peau humaine in vivo via l’IRM. (Université de Technologie de Compiègne, 2007).

[26] Pierard, G. E. & Lapi, C. M. Physiopathological variations in the mechanical properties of skin. *Arch. Derm. Res.* 231–239 (1977). 10.1007/BF00561418 603255

[27] Groves, R. B. Quantifying the mechanical properties of skin in vivo and ex vivo to optimise microneedle device design. (Cardiff University, 2011). 10.1080/10255842.2011.596481 21749225

[28] Annaidh, A. N., Bruyère, K., Destrade, M., Gilchrist, M. D. & Ottenio, M. Characterization of the anisotropic mechanical properties of excised human skin. *J. Mech. Behav. Biomed. Mater.* **5**, 139–148 (2012). 22100088 10.1016/j.jmbbm.2011.08.016

[29] Gerin, G. Validation de l’utilisation d’un extensomètre uni-axial pour l’étude des propriétés mécaniques de la peau du chien. (Université Claude Bernard de Lyon, 2012).

[30] Prete, Z. D., Antoniucci, S., Hoffman, A. H. & Grigg, P. Viscoelastic properties of skin in Mov-13 and Tsk mice. *J. Biomech.* (2004). 10.1016/j.jbiomech.2004.01.015 15336923

[31] Gahagnon, S. Étude in vivo du comportement mécanique du derme par une méthode élastographique haute résolution: applications à l’exploration d’anomalies du tissu élastique (syndrome de Marfan). (Université François Rabelais de Tours, 2009).

[32] Agache, P. G., Monneur, C. & Leveque, J. L. Mechanical properties and Young’s modulus of human skin in vivo. *Arch. Dermatol. Res.* **269**, 221–232 (1980). 7235730 10.1007/BF00406415

[33] Aubert, L., Anthoine, P., De Rigal, J. & Leveque, J. L. An in vivo assessment of the biomechanical properties of human skin modifications under the influence of cosmetic products. *Int. J. Cosmet. Sci.* **7**, 51–59 (1985). 19460014 10.1111/j.1467-2494.1985.tb00396.x

[34] Escoffier, C. et al. Age-Related Mechanical Properties of Human Skin: An In Vivo Study. *J. Invest. Dermatol.* **93**, 353–357 (1989). 2768836

[35] Edwards, C. & Marks, R. Evaluation of Biomechanical Properties of Human Skin. *Clin. Dermatol.* **13**, 375–380 (1995). 8665446 10.1016/0738-081x(95)00078-t

[36] Hendriks, F. M., Brokken, D., Oomens, C. W. J., Bader, D. L. & Baaijens, F. P. T. The relative contributions of different skin layers to the mechanical behavior of human skin in vivo using suction experiments. *Med. Eng. Phys.* 28, 259–266 (2006). 10.1016/j.medengphy.2005.07.001 16099191

[37] Dobrev, H. Application of Cutometer area parameters for the study of human skin fatigue. *Skin. Res. Technol.* **11**, 120–122 (2005). 15807810 10.1111/j.1600-0846.2005.00090.x

[38] Gniadecka, M. Skin mechanical properties present adaptation to man’s upright position. 188–190 (1974). 10.2340/0001555574188190 7915458

[39] Stroumza, N., Bosc, R., Hersant, B., Hermeziu, O. & Meningaud, J. P. Intérêt du cutomètre pour l’évaluation de l’efficacité des traitements cutanés en chirurgie plastique et maxillo-faciale. *Rev. Stomatol. Chir. Maxillo-Faciale Chir. Orale*. **116**, 77–81 (2015). 10.1016/j.revsto.2015.02.002 25817308

[40] Rosado, C., Antunes, F., Barbosa, R., Fernando, R. & Rodrigues, L. M. Cutiscan^®^ - A new system of biomechanical evaluation of the skin in vivo - comparative study of use depending on the anatomical site. *Biomed. Biopharm. Res.* **12**, 49–57 (2015).

[41] Hendriks, F. Mechanical behaviour of human epidermal and dermal layers in vivo. (Technische Universiteit Eindhoven, 2005).

[42] Jachowicz, J., McMullen, R. & Prettypaul, D. Indentometric analysis of in vivo skin and comparison with artificial skin models. *Skin Res. Technol.* 13, 299–309 (2007). 10.1111/j.1600-0846.2007.00229.x 17610652

[43] Zahouani, H., Boyer, G., Pailler-Mattei, C., BenTkaya, M. & Vargiolu, M. Effect of human ageing on skin rheology and tribology. *Wear* **271**, 2364–2369 (2011).

[44] Boyer, G. et al. Non contact method for in vivo assessment of skin mechanical properties for assessing effect of ageing. *Med. Eng. Phys.* **34**, 172–178 (2012). 21807547 10.1016/j.medengphy.2011.07.007

[45] Pailler-Mattei, C. & Zahouani, H. Study of adhesion forces and mechanical properties of human skin in vivo. *J. Adhes. Sci. Technol.* **18**, 1739–1758 (2012).

[46] Kearney, E. M. et al. Evaluation of skin firmness by the DynaSKIN, a novel non-contact compression device, and its use in revealing the efficacy of a skincare regimen featuring a novel anti-ageing ingredient, acetyl aspartic acid. *Skin Res. Technol.* 0, 1–14 (2016). 10.1111/srt.12314 27546316

[47] Derail, C., Ehrenfeld, F., Laffore, A., Nardin, C. & Blanchard, B. Système de mesure des propriétés mécaniques d’un échantillon de peau. Patent WO 2022/129813 A1. OMPI (2022).

[48] Holt, B., Tripathi, A. & Morgan, J. Viscoelastic Response of Human Skin to Low Magnitude Physiologically Relevant Shear. *J. Biomech.* **28**, 2689–2695 (2008). 10.1016/j.jbiomech.2008.06.008 PMC2584606 18672246

[49] Geerligs, M., Peters, G., Ackermans, P., Oomens, C. & Baaijens, F. Linear viscoelastic behavior of subcutaneous adipose tissue. *Biorheology* **45**, 677–688 (2008). 19065014

[50] Griffin, M. F., Leung, C., Premakumar, Y., Szarko, M. & Butler, P. E. Comparison of the mechanical properties of different skin sites for auricular and nasal reconstruction. *J. Otolaryngol. Head Neck Surg.* (2017). 10.1186/s40463-017-0210-6 PMC5395887 28420435

[51] Khatyr, F., Imberdis, C., Vescovo, P., Varchon, D. & Lagarde, J.-M. Model of the viscoelastic behaviour of skin in vivo and study of anisotropy. *Skin Res. Technol.* 10, 96–103 (2004). 10.1111/j.1600-0846.2004.00057.x 15059176

[52] Ottenio, M., Tran, D., Annaidh, A. N., Gilchrist, M. D. & Bruyère, K. Strain rate and anisotropy effects on the tensile failure characteristics of human skin. *J. Mech. Behav. Biomed. Mater.* 41, 241–250 (2015). 10.1016/j.jmbbm.2014.10.006 25455608

[53] Holzapfel, G. A. Biomechanics of Soft Tissue. *Comput. Biomech.* (2000).

[54] Tong, P. & Fung, Y. The Stress-strain relationship for the skin. *J. Biomech.* **9**, 649–657 (1976). 965417 10.1016/0021-9290(76)90107-x

[55] Iivarinen, J. T., Korhonen, R. K., Julkunen, P. & Jurvelin, J. S. Experimental and computational analysis of soft tissue mechanical response under negative pressure in forearm. *Skin Res. Technol.* 19, 356–365 (2013). 10.1111/j.1600-0846.2012.00652.x 22650760

[56] Diani, J., Fayolle, B. & Gilormini, P. A review on the Mullins effect. *Eur. Polym. J.* 45, 601–612 (2009).

[57] Eurofins BIO-EC. https://www.bio-ec.fr/ex-vivo/.

[58] Liu, Z. & Yeung, K. The preconditioning and stress relaxation of skin tissue. *J. Biomed. Eng.* 2, 22–28 (2008).

